# The effect of organ-specific tumor microenvironments on response patterns to immunotherapy

**DOI:** 10.3389/fimmu.2022.1030147

**Published:** 2022-11-17

**Authors:** Jordan W. Conway, Jorja Braden, James S. Wilmott, Richard A. Scolyer, Georgina V. Long, Inês Pires da Silva

**Affiliations:** ^1^ Melanoma Institute Australia, The University of Sydney, Sydney, NSW, Australia; ^2^ Faculty of Medicine and Health, The University of Sydney, Sydney, NSW, Australia; ^3^ Charles Perkins Centre, The University of Sydney, Sydney, NSW, Australia; ^4^ Department of Tissue Pathology and Diagnostic Oncology, Royal Prince Alfred Hospital and NSW Health Pathology, Sydney, NSW, Australia; ^5^ Royal North Shore Hospital, Sydney, NSW, Australia; ^6^ Mater Hospital, Sydney, NSW, Australia; ^7^ Westmead and Blacktown Hospitals, Sydney, NSW, Australia

**Keywords:** immunotherapy, metastasis, tumor microenvironment, organ-specific immune response, organ-specific immune microenvironment

## Abstract

Immunotherapy, particularly immune checkpoint inhibitors, have become widely used in various settings across many different cancer types in recent years. Whilst patients are often treated on the basis of the primary cancer type and clinical stage, recent studies have highlighted disparity in response to immune checkpoint inhibitors at different sites of metastasis, and their impact on overall response and survival. Studies exploring the tumor immune microenvironment at different organ sites have provided insights into the immune-related mechanisms behind organ-specific patterns of response to immunotherapy. In this review, we aimed to highlight the key learnings from clinical studies across various cancers including melanoma, lung cancer, renal cell carcinoma, colorectal cancer, breast cancer and others, assessing the association of site of metastasis and response to immune checkpoint inhibitors. We also summarize the key clinical and pre-clinical findings from studies exploring the immune microenvironment of specific sites of metastasis. Ultimately, further characterization of the tumor immune microenvironment at different metastatic sites, and understanding the biological drivers of these differences, may identify organ-specific mechanisms of resistance, which will lead to more personalized treatment approaches for patients with innate or acquired resistance to immunotherapy.

## 1 Introduction

Despite significant success with immunotherapy across different cancers, the majority of patients with advanced disease will either not respond (innate resistance) or develop resistance after initial response (acquired resistance) to immune checkpoint inhibitors. The presence versus absence of specific sites of metastatic disease has been associated with differences in both local and overall responses to immunotherapy, as well as in progression-free and overall survival. Less frequently, patients with multiple sites of disease may present with a heterogenous response, where some metastases may respond whilst others continue to progress, commonly referred to clinically as a ‘mixed response’. These observations suggest that there are likely organ-specific mechanisms of response and resistance to immune checkpoint inhibitors, which should be taken into consideration when choosing therapy for individual patients.

Advances in single-cell multi-omic technologies has allowed for a more in-depth exploration and characterization of different immune cell types and immune cell subsets (1, 2). These technologies and studies unveil the inter and intratumoral heterogeneity, and the association with immunotherapy responses. Increased awareness of the tumor immune microenvironment at different metastatic sites is key to better understand the role this plays in local and systemic responses when treated with immunotherapy. Furthermore, such understanding will likely uncover new strategies to more effectively tailor the application of immunotherapies and novel treatments.

This review aims to summarize data regarding the organ-specific tumor microenvironment and the respective response patterns to immunotherapy across different cancer types. This review further aims to highlight opportunities to apply this understanding to develop more effective treatment strategies and improve outcomes for cancer patients.

## 2 Standard immune checkpoint inhibitors

### 2.1 Mechanisms of action of standard immune checkpoint inhibitors

The immune checkpoints, programmed cell death protein 1 (PD-1) and Cytotoxic T-lymphocyte-associated protein 4 (CTLA-4), both act to negatively regulate the immune system and are key facilitators of immune homeostasis and prevention of autoimmunity.

#### 2.1.1 Anti-programmed cell death protein 1

Expression of PD-1 is typically induced by T-cell receptor (TCR) stimulation and is expressed on a variety of immune cells, particularly on activated T cells and regulatory T cells (Tregs) but has also been shown to be expressed on B cells, monocytes, natural killer (NK) cells and dendritic cells (3–5). Chronic exposure to antigens leads to an over-expression of PD-1 on T cells, which has been used as an indicator of T cell exhaustion (6). PD-1 has 2 natural ligands, PD-L1 and PD-L2. Antigen presenting cells such as B cells, dendritic cells and macrophages, often express both PD-L1 and PD-L2, but expression of PD-L1 and PD-L2 can also be induced on hematopoietic and non-hematopoietic cells including endothelial cells, through pro-inflammatory cytokines like interferon gamma (IFNγ) (3). Host tissue expression of PD-L1/PD-L2 has therefore been shown to increase during an inflammatory response (3, 5, 7, 8). As an important immune tolerance mechanism to prevent auto-immunity, PD-1 binding to PD-L1/PD-L2 leads to reduced T cell proliferation, reduced cytotoxic activity, and a decreased ability to produce cytokines (9, 10). Tumors, including melanoma and non-small cell lung cancer (NSCLC), utilize this PD-1/PD-L1 axis to suppress and evade the host immune response (3, 11, 12). Some tumors express PD-L1 to varying degrees, although the mechanism of cancer immune tolerance is thought to primarily occur within the tumor microenvironment *via* cytokine-induced expression of PD-L1 on tumor cells (3). This local activity within the TME is thought to be the contributor to the high efficacy of treatments (anti-PD-1 and anti-PD-L1) in advanced cancer patients. Immune checkpoint inhibiting antibodies, specific to either PD-1 or PD-L1, block this interaction and have become the standard of care therapies across various cancer types.

#### 2.1.2 Anti-cytotoxic T-lymphocyte-associated protein 4

Unlike PD-1, which acts at later stages of T cell activation and typically in the tumor microenvironment, CTLA-4, another immune checkpoint, acts earlier in the immunity cycle. CTLA-4 is thought to act during T cell priming and activation, typically in regional lymph nodes (13–15). CTLA-4 is often expressed on Tregs and contributes to their inhibitory function which helps maintain immune self-tolerance. However, expression of CTLA-4 is also induced on T cells upon early activation (16). CTLA-4 and CD28, a T cell co-stimulatory molecule, are homologs and competitively bind to their ligands, CD80 and CD86 (B7 family), which are expressed on antigen presenting cells (13, 17). Recognition of antigen expression induces TCR activation and CD28 binding to CD80/CD86, which leads to increased T cell proliferation and IL-2 production (13). In contrast, CTLA-4 has a greater binding affinity for CD80/CD86, and when it binds to the ligands (instead of CD28), causes reduction of T cell proliferation and survival, and reduced IL-2 production, suppressing the host immune response (13, 17, 18). Therefore, CTLA-4 blockade enhances T cell anti-tumor response and consequently improves clinical outcomes in some cancer types.

### 2.2 Clinical applications of immune check point inhibitors for cancer patients

Immune checkpoint inhibitors such as anti-PD-1 (nivolumab, pembrolizumab, cemiplimab), anti-PD-L1 (durvalumab, atezolizumab) and anti-CTLA-4 (ipilimumab [IPI]) were shown to significantly improve survival in melanoma and other cancers, including non-melanoma skin cancers (merkel cell carcinoma and cutaneous squamous cell carcinoma), lung cancer, renal cell carcinoma, head and neck cancer, and hepatocellular carcinoma.

#### 2.2.1 Melanoma

In AJCC (American Joint Committee on Cancer) stage IV melanoma patients, anti-PD-1 monotherapy or in combination with IPI induces unprecedented long-term responses in a subset of patients, with a 36% and 29% progression-free survival (PFS) rate at 5 years, and with 44% and 52% overall survival (OS) rate at 5 years, for anti-PD-1 or IPI+anti-PD-1, respectively (19). Immune checkpoint inhibitors have become the standard of care for patients with advanced melanoma, as well as for resected stage III melanoma in the adjuvant setting (20–23), with an associated reduction in the risk of recurrence (HR 0.60, p<0.0001) (23). Moreover, immune checkpoint inhibitors have also shown impressive results in the adjuvant setting for high-risk stage II (stage IIB/C) and in the neoadjuvant setting for resectable stage III/IV melanoma (24–26).

#### 2.2.2 Lung cancer

In metastatic non-small cell lung cancer (NSCLC), anti-PD-1 can be used as monotherapy or in combination with chemotherapy, depending on PD-L1+ tumor expression, patient characteristics, amongst other variables. The phase 3 trial KEYNOTE-024 showed improved OS (median OS, 30 months versus 14.2 months) and PFS (median PFS, 10.3 months versus 6 months) in advanced NSCLC patients treated with first-line anti-PD-1 monotherapy compared to platinum-based chemotherapy in patients with tumors with a PD-L1 tumor proportion score of 50% or greater (27, 28). In addition, the KEYNOTE-189 (non-squamous NSCLC) and the KEYNOTE-407 (squamous NSCLC) trials showed that the addition of anti-PD-1 to a platinum-doublet chemotherapy improved OS (hazard ratio [HR], 0.56; 95% CI: 0.45 to 0.70 and [HR], 0.64; 95% CI: 0.49−0.85, respectively) and PFS (hazard ratio [HR], 0.48; 95% CI: 0.40 to 0.58 and [HR], 0.56; 95% CI: 0.45−0.70, respectively) compared to chemotherapy alone regardless of PD-L1 expression (29, 30). Platinum-doublet chemotherapy with anti-PD-1 is now the standard first line treatment for metastatic NSCLC patients whose tumor lacks an actionable mutation. In patients with PD-L1 positive (PD-L1 1% or more) NSCLC, the combination of IPI+anti-PD-1 has also shown better OS compared to chemotherapy (median OS, 17.1 months versus 14.9 months) (31), and IPI+anti-PD-1 combined with chemotherapy (2 cycles) showed superior overall survival compared to chemotherapy alone, regardless of PD-L1 expression (median OS, 15.6 months versus 10.9 months) (32). Whether IPI+anti-PD-1 is also superior to chemotherapy + PD-1 in first line for NSCLC is unknown. Anti-PD-1 monotherapy has also shown survival advantage (PFS and OS) compared to docetaxel in second line setting, after failing first line platinum based doublet (without anti-PD-1), regardless of PD-L1 expression (33–35). Also, consolidation with anti-PD-L1 after concurrent chemoradiotherapy has significantly reduced the risk of recurrence for locally advanced stage III NSCLC and it is established as standard of care in this setting (36). Recent studies have also shown promising results with the addition of anti-PD-1 in both adjuvant (37, 38) and neoadjuvant (39, 40) settings, but more mature data is needed to confirm these results.

#### 2.2.3 Other cancers

In renal cell carcinoma, the combination of IPI+anti-PD-1 was associated with better response (objective response rate [ORR] 42% versus 27%) and PFS (median PFS, 11.6 versus 8.4 months) compared to sunitinib in patients with intermediate or poor risk renal cell carcinoma (RCC) in the first line setting (41). Anti-PD-1 monotherapy was also associated with better survival (PFS and OS) compared with everolimus (mTOR inhibitor) in patients with clear-cell RCC after failing one or two lines of anti-angiogenic agents (42). In head and neck squamous cell carcinoma, anti-PD-1 monotherapy was associated with superior overall survival compared to second line standard single agent therapy (Cetuximab, docetaxel, or methotrexate) (median OS, 7.1 months versus 5.1 months), after progressing within 6 months of platinum-based chemotherapy (43). Also, in patients with unresectable hepatocellular carcinoma, anti-PD-L1 in combination with anti-VEGF demonstrated improved PFS and OS compared to sorafenib (median PFS, 6.8 months versus 4.3 months; and OS at 1 year, 67.2% versus 54.6%) (44). Anti-PD-1 has also demonstrated activity in the second line setting in patients with metastatic HCC progressing on sorafenib (45–48). Anti-PD-1 monotherapy is also frequently used in thyroid cancer. However, due to lower mutation burden of some thyroid cancers and their immunosuppressive tumor microenvironment, results seen in cancers such as melanoma, lung cancer and RCC have not been matched in thyroid cancer. Studies assessing the combination of anti-PD-1 in conjunction with anti-CTLA-4, with BRAF inhibitors, as well as, anti-VEGF agents are ongoing particularly for patients with advanced thyroid cancer aiming to improve response to anti-PD-1 therapy (49, 50).

Whilst immune checkpoint inhibitors became the standard of care for melanoma, NSCLC, RCC, head and neck, and hepatocellular carcinoma, many other cancer types are still in the early stages of evaluating the efficacy of these treatments, most of which are limited to clinical trials only or for a select subset of patients, e.g. Triple negative breast cancer (TNBC) and Microsatellite Instability high colorectal cancer (51–55).

## 3 Site specific clinical outcomes and the tumor microenvironment

### 3.1 Liver metastases

#### 3.1.1 Clinical outcomes in patients with liver metastases treated with immunotherapy

The presence of liver metastases is a significant prognostic factor and has been associated with reduced survival rates in cancer patients (56, 57). Overall, across all cancers, patients with liver metastases also have worse outcomes than those without liver metastases when treated with anti-PD-1 based immunotherapy. A summary of clinical studies assessing the local and systemic response of liver metastases to immunotherapy are summarized in **Table 1**.

**Table 1:** Summary of the organ specific response and survival rates from highlighted clinical studies.

Trial/Study	Trial/Study type	Cancer types	Immunotherapy treatment	Number of patients	Sites of metastasis				
					Liver	Lung	Brain	Bone	Others
Tournoy et al.	Retrospective	NSCLC	Nivolumab	267	↓ OS			Reduced OS	
Goldberg et al.	Clinical trial	NSCLC	Pembrolizumab	42			ORR achieved ORR: PD-L1+ > ORR PD-L1- tumors		
Liao et al.	Retrospective	NSCLC	Nivolumab	70			OS benefit OS: PD1+ WBRT > OS : WBRT		
Schmid et al.	Retrospective	NSCLC	Nivolumab	52					LN: ↑ OSRR
Si-cong ma et al.	Secondary analysis: OAK trial	NSCLC	PDL1 vs. chemotherapy	425					Adrenal gland: ↑ OS
Adachi et al.	Retrospective	NSCLC (SCC/ADC)	Nivolumab	296	↓ PFS and ↓ OS				
Landi et al.	Retrospective	NSCLC (Squamous and non-squamous)	Nivolumab	1588				↓ ORR, ↓ PFS and ↓ OS	
Li et al.	Retrospective	NSCLC (Squamous and non-squamous)	Multiple ICIs	204				↓ OS and ↓ PFS	
Topalian et al.	Clinical trial: Checkmate 003	NSCLC, Melanoma, Renal cell carcinoma	Nivolumab	270				↓OS	
Tumeh et al.	Secondary analysis: Keynote 001, Keynote 002, Keynote 006	Melanoma and NSCLC	Pembrolizumab	336 (melanoma), 165 (NSCLC)	↓ ORR and ↓ PFS	↑ PFS			
Yu et al.	Retrospective	Melanoma and NSCLC	IPI+PD1	182 (melanoma), 279 (NSCLC)	↓ OS and ↓ PFS				
Joseph et al.	Secondary analysis: Keynote 001	Melanoma	Pembrolizumab	583	↓ ORR and 1-year OS	↑ ORR and ↑ 1-year OS			
Pires da Silva et al.	Retrospective	Melanoma	IPI+PD1	140	↓ OSRR, ↓ ORR, ↓ OS and PFS	↑ ORR and ↑ PFS		↓ ORR and ↓ PFS	Spleen: ↓ ORR, ↓ OFS and ↓ OS. GI and soft-tissue: ↑ OSRR
Long et al.	Clinical trial: ABC trial	Melanoma	PD1 or IPI+PD1	79			ORR achieved ORR: IPI+PD1 > ORR: PD1		
Tawbi et al.	Clinical trial: Checkmate 204	Melanoma	IPI+PD1	165			ORR achieved ORR: Asymptomatic > ORR: symptomatic		
Borgers et al.	Retrospective	Melanoma	Multiple ICIs	168					Adrenal gland: ↓ DCR and ↓ OS
Beer et al.	Clinical trial: CA184-095	Castrate resistant prostate cancer	IPI *vs.* placebo	399				↓ OS	
Fukuoka et al.	Secondary analysis: REGONIVO	Gastric cancer/Colorectal carcinoma	Nivolumab + Regorafenib	50	↓ ORR	↑ ORR			
Wang et al.	Retrospective	MSS colorectal cancer	PD1/PDL1	95	↓ ORR and ↓ PFS				
lu et al	Retrospective	Hepatocellular carcinoma	PD1, PDL1, CTLA4 or IPI+PD1	75	↓ OSRR	↑ ORR			
Negishi et al.	Retrospective	Renal cell carcinoma	Nivolumab	68			↓ ORR		
Escudier et al.	Clinical trial: Checkmate 025	Renal cell carcinoma	Nivolumab + Everolimus	146				OS benefit OS PD1 > OS everolimus	
Emans et al.	Retrospective	Triple negative breast cancer	Atezolizumab (PDL1)	116	↓ ORR and ↓ OS and PFS				
Adams et al.	Secondary analysis: Keynote 086	Triple negative breast cancer	Pembrolizumab	170	↓ ORR				LN: ↑ ORR

↓ = reduced/shorter, ↑ = greater/longer, > = greater than.

In a retrospective study of 140 metastatic melanoma patients, liver metastases had reduced local response rates compared with other sites of disease including lung, subcutaneous, soft-tissue, and gastrointestinal metastases when treated with combination IPI+anti-PD-1 in the first line setting (58). On multivariate analysis, it was shown that the presence of liver metastases was significantly associated with reduced overall response rate (ORR, 43.6% versus 76.8%), shorter progression-free survival (6-month PFS, 43% versus 80%) and overall survival (1-year OS, 65% versus 94%) compared to those patients without liver metastases (58). Remarkably, this difference in clinical outcomes between patients with and without liver metastases was not seen in patients treated with targeted therapy (BRAF+MEK inhibitors) (59), suggesting resistance in the liver is specific to immunotherapy. Tumeh et al. also demonstrated that in both the discovery (KEYNOTE-001 trial) and validation (KEYNOTE-002 and KEYNOTE-006 trials) melanoma cohorts, patients with liver metastases had reduced PFS (median PFS, 5.1 months in the discovery cohort and 2.7 months in the validation cohort) compared to patients without liver metastases (median PFS, 20.1 months in the discovery cohort and 18.5 months in the validation cohort) (60).

A retrospective study of NSCLC patients comprised of both squamous cell carcinoma (SCC) and non-squamous cell carcinoma (adenocarcinoma [ADC]) treated with anti-PD-1 monotherapy showed that patients with liver metastases have shorter PFS and OS compared with patients without liver metastases (hazard ratio [HR] for PFS = 1.62, 95% CI, 1.11-2.36; HR for OS = 1.62, 95% CI, 1.09-2.41) (61). In another large retrospective study of almost 24,000 patients, including patients with NSCLC (ADC and SCC) and small cell lung cancer (SCLC), for those with a solitary metastatic site, it was shown that patients with liver metastases had significantly poorer outcomes compared to patients with other solitary metastatic sites, including brain and bone. In addition, a different study which included metastatic ADC or extensive-stage SCLC with multiple sites of disease, those with liver metastases had shorter overall survival compared to those without liver metastases (median OS for ADC 3 *vs.* 4 months, and median OS for SCLC 4 *vs.* 6 months, patients without versus with liver metastases, respectively) (57). This difference was also observed in the KEYNOTE-001 trial, a phase 1 clinical trial that included patients with progressive locally advanced or metastatic carcinoma, melanoma, or NSCLC. Patients with metastatic NSCLC with liver metastasis had reduced PFS compared to patients without liver metastases (median PFS, 1.8 months *vs.* 4.0 months) (60).

A retrospective study by Lu et al. including advanced hepatocellular carcinoma patients treated with either single agent anti-PD-(L)1, IPI or the combination IPI+anti-PD-1, also observed differences in organ specific response. Specifically, they found that the primary liver lesions had the worst response compared to any metastatic site (62). Furthermore, in the subset of patients with multiple measurable sites of disease there was discordant response in 75% of patients, with extra hepatic tumor response whilst progressing in the liver. In another subgroup analysis of patients with only dual lung and liver metastases, half of the patients progressed in the liver whilst achieving a response in the lung metastases, whilst there were no patients who achieved a response in the liver but progressed in the lung (62).

Whilst immunotherapy is often used for microsatellite instable (MSI) colorectal cancer, a study by Wang et al. in microsatellite stable (MSS) colorectal cancer showed that no patients with liver metastases had a response to anti-PD-(L)1 therapy versus an ORR of 19.5% in patients without liver metastases. Furthermore, patients with liver metastases had a shorter PFS compared to those without liver metastases (63). Similarly, in a phase 1 study (NCT01375842) of TNBC patients treated with PDL1, those with liver metastases had poorer response and survival compared to those without liver metastases (64). Likewise, the KEYNOTE-086 trial, which included previously treated metastatic TNBC patients, showed no objective response in patients treated with anti-PD-1 that had liver metastases, while 7.3% of those without liver metastases responded to treatment (65).

#### 3.1.2 Liver-specific tumor microenvironment

The liver receives blood supply directly from the gastrointestinal tract and is constantly exposed to various microbes and pathogens (66). The liver is therefore one of the first lines of immune defense against foreign pathogens, with site specific immune cell populations tasked with maintaining immune homeostasis and protection. Hepatocytes are the primary parenchymal cells of the liver and help activate the innate immune response (67). Specialized liver sinusoidal endothelial cells not only act as a barrier, but also have various functions including metabolism and defense against invading pathogens through antigen presentation and endocytic capabilities (68, 69). Kupffer cells are also a unique tissue resident liver macrophage population which play a role in immune defense, promoting inflammation, regulating metabolism, and directly traveling along the liver sinusoids to phagocytose pathogens (70, 71).

Hepatocellular carcinoma (HCC), as a primary tumor arising from the liver has been shown to harbor an immunosuppressive tumor microenvironment. Increased expression of pro-tumoral cytokines and chemokines including IL-8, TGF- β and CXCL12, infiltration of immunosuppressive cells including myeloid-derived suppressor cells and tumor-associated macrophages, as well as, increased expression of immune checkpoints including TIM-3 have been associated with reduced survival rates in treatment naïve patients (72).

**Figure 1:**
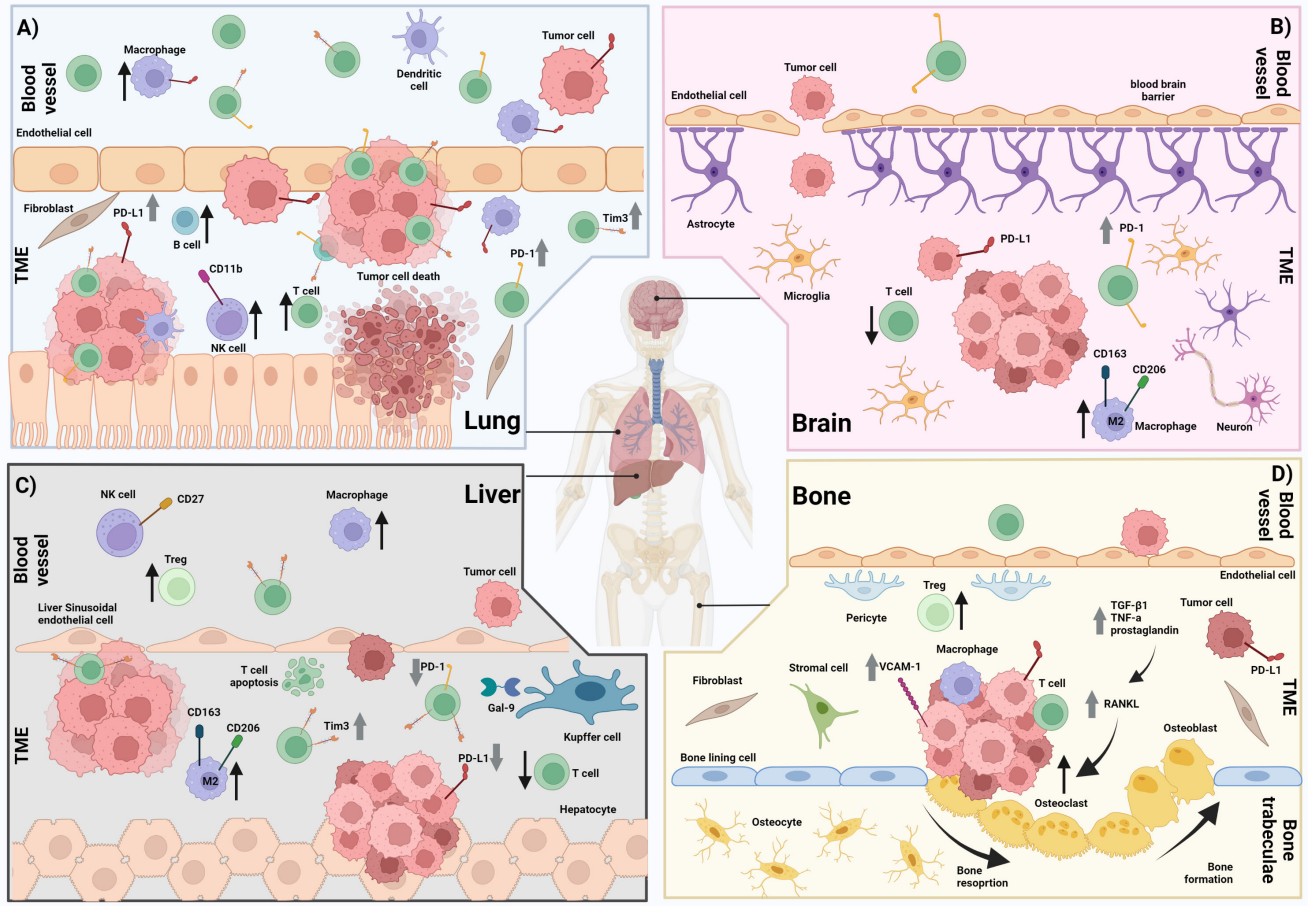
The immune microenvironment in lung, brain, liver and bone metastases. **(A)** Lung metastasis showing a higher infiltration of T cells, macrophages, dendritic cells, B cells, fibroblasts and CD11b+ NK cells compared with other sites of metastases (e.g. liver, brain, and bone). There is increased T cell expression of PD-1 and increased tumor and macrophage expression of PD-L1 compared with other sites of metastases (e.g. liver, and brain). **B)** Brain metastasis showing a higher infiltration of M2 macrophages and reduced infiltration of T cells compared with other metastases (e.g. lung). There is also increased T cell expression of PD-1 compared with other metastases (e.g. liver) and a disrupted blood brain barrier. **(C)** Liver metastasis showing reduced infiltration of T cells but increased infiltration of macrophages including M2 macrophages, and CD27+ NK cells compared with other metastases (e.g. lung and bone) There is also reduced T cell expression of PD-1 and high T cell expression of Tim-3 compared with other metastases (e.g. brain, lung, and bone). Increased T cell apoptosis has been observed. **(D)** Bone metastasis showing an increased infiltration of fibroblasts and stromal cells compared with other metastases (e.g. liver and brain). There is also increased tumor expression of PD-L1 and VCAM-1 compared with other metastases (e.g. liver and brain). An increase in the RANKL pathway leading to increased osteoclasts and subsequently increased osteoclast driven bone resorption is also observed in bone metastases. ↑ = Increased cell infiltration ↓ = Decreased cell infiltration ⇧ = Increased expression ⇩ = Decreased expression TME = Tumor microenvironment. Created with BioRender.com.

Liver metastases overall have been shown to be less immunogenic compared to other sites of metastases. They are characterized by reduced immune cell infiltration, specific immune cell profiles and upregulation of immunosuppressive mechanisms. An overview of the tumor immune microenvironment of liver metastases from reported studies has been depicted in **Figure 1**.

Metastases that are established in the liver harbor a unique tumor microenvironment. Our group has recently shown that melanoma liver metastases had reduced numbers of CD3+ T cells and that these cells were also further away from melanoma cells, particularly compared to lung and lymph node metastases (73). Furthermore, liver metastases harbored fewer T cells expressing PD-1 whilst having a higher proportion of T cells expressing the immune checkpoint T cell immunoglobulin domain and mucin domain 3 (TIM-3). Liver metastases also had reduced expression of PD-L1 on both tumor cells and macrophages despite having an increased density of macrophages compared to other sites (73). Tumeh et al. also explored the tumor microenvironment of melanoma liver metastases and, similarly, found that liver metastases had reduced CD8+ T cells density compared to other sites of melanoma metastasis. Moreover, a study on an *in vivo* melanoma model showed that, tumors established in the liver had lower mRNA and protein expression of IL-8 compared to subcutaneous and lung metastases, suggesting local modulation within specific organ sites (74).

A study conducted by Ballas and colleagues using a C57BL/6 mouse model found that B16.F1 melanoma cell line injected intravenously metastasized preferentially to the lung, and not to the liver (75). However, depletion of NK cells upon PK136 injection increased the metastatic potential for the liver. The authors also observed that liver metastases in the murine model had an increased density of CD27+ CD11b- NK cells, thought to represent an immature subset of NK cells. Further adoptive transfer experiments of liver NK cell subsets suggested that the specific subset of immature NK cells, characterized as CD27+ CD11b-, may play a protective role in the development of liver metastases but not metastases at other sites (75).

The presence of liver metastases may also modulate the systemic anti-tumor immune response at other sites of metastasis. In the study by Tumeh et al, patients with liver metastases also had reduced CD8+ T cells at distant sites compared to patients without liver metastases (60). Yu and Green et al. have generated *in vivo* models with one or two concurrent sites of metastases by injecting subcutaneously, intrahepatically or intrasplenically melanoma (B16F10), colon adenocarcinoma (MC38) and pancreatic ducal adenocarcinoma (KPC2) cell lines, to assess response to anti-PD-L1 treatment and changes in the tumor immune microenvironment (59). They observed that the presence of MC38 liver tumors reduced the ability of PD-L1 based therapy to increase the infiltration of CD8+ T cells in concurrent subcutaneous tumors. These findings suggest that liver metastases were able to siphon T cells from other sites and from systemic blood circulation. A reduced number of antigen specific T cells, as indicated by tetramer staining, was observed in extrahepatic sites in mice with concurrent liver metastases compared to those without liver metastases. An increase in T cells was subsequently observed in the respective liver tumors (59). Furthermore, there was an increase in T cell apoptosis in liver metastases versus metastases at other sites, suggesting that the T cells siphoned from the systemic circulation and other organs then undergo apoptosis in the liver (59). This study also showed that there is an increase of Cd11b+F4/80+ myeloid cells and reduced CD4+ T cells in the subcutaneous tumors in the presence versus absence of liver metastases. Furthermore single-cell RNA sequencing of the normal livers in mice with subcutaneous only MC38 tumors and of the livers of mice with concurrent MC38 liver metastases showed an enrichment for M2-like macrophage immunosuppressive gene signature and a reduced M1-like macrophage inflammatory gene signature on monocyte derived macrophages in the liver tumor bearing mice. Lee et al. reported a similar study where C57BL/6 mice were implanted with MC38 tumors cells subcutaneously only or subcutaneously concurrently with subcapsular or hemispleen injection to obtain liver tumors. Mice were then treated with anti-PD-1 monotherapy, and it was observed that those with concurrent liver and subcutaneous tumors had reduced survival and were not as responsive to therapy as those with subcutaneous metastases only (76). Assessment of the tumor immune infiltrate showed that subcutaneous tumors of mice with liver metastases had reduced CD8+ T cell expression of activation markers including ICOS, PD-1 and CTLA-4, as well as expression of intracellular IFNγ (76). Subcutaneous tumors showed an increased infiltration of CD11b+ myeloid derived suppressor cells (MDSC) in mice with concurrent liver metastases, but not in mice with concurrent metastases in other anatomical sites (76). In this study, researchers hypothesized that Tregs were responsible for the immunosuppressive properties of liver metastases. Diphtheria toxin administered to deplete Tregs showed an enhanced tumor rejection in mice with liver metastases and an increase in the expression of IFNγ, ICOS and CD107a on CD8+ T cells. Further treatment with the anti-CTLA-4 clone 9H10 to deplete Tregs in addition to anti-PD-1 saw a complete response at both the subcutaneous and liver tumors in mice with two-site tumor (76). However, whilst this study was undertaken in a murine model this mechanism of complete response in all patients has not been able to be replicated in humans treated with combination IPI+anti-PD-1 (19).

### 3.2 Lung metastases

#### 3.2.1 Clinical outcomes in patients with lung metastases treated with immunotherapy

Whilst there is more limited clinical data available, in contrast to liver metastases, the presence of lung metastases has been associated with good responses when treated with anti-PD-1 based immunotherapy. A summary of clinical studies assessing the local and systemic response of lung metastases are summarized in **Table 1**.

The KEYNOTE-001 trial also showed that melanoma patients with lung metastases only, had better ORR (62%) and survival (1-year OS of 89%) compared to those patients with other sites of disease, particularly those with liver metastases only (ORR 22% and 1-year OS of 53%), as mentioned above (77). In addition, we recently showed that melanoma lung metastases had a higher organ-specific response rate (OSRR) compared to other sites of metastases, including liver when treated with combination IPI+anti-PD-1 in the first line setting. Moreover, the presence of lung metastases was significantly associated with better ORR (Odds ratio [OR] 2.68) and a longer PFS (HR 0.46) compared to patients without lung metastases (58). More recently, a large international multi-center study led by our group developed a predictive tool for anti-PD-1+/-IPI, and identified lung metastases as a predictor of good response to these treatment in advanced melanoma patients (78).

In a study by Lu et al. looking at advanced hepatocellular carcinoma patients treated with single agent anti-PD-1 or combination IPI+anti-PD-1, those with lung metastases had the greatest response rates (62). Also, in a phase 1 trial of advanced gastric or colorectal cancer patients treated with Regorafenib (Anti-VEGF) plus anti-PD-1, colorectal cancer patients with lung metastases had a higher response rate (ORR, 66.7%) particularly compared to patients with liver metastases (ORR, 40%) (79).

#### 3.2.2 Lung-specific tumor microenvironment

Similar to the liver, the lung acts as a first line of defense against inhaled pathogens and other exogenous substances that enter the body *via* the airway. Unique epithelial cells in the lung, not only provide a physical barrier, but also aid in antimicrobial peptide production and inflammation (80). Furthermore, like Kupffer cells in the liver, the lung harbors unique alveolar macrophages which help protect against pathogens and surfactant removal. Alveolar macrophages typically act near the epithelium where they are able to come into direct contact with pathogens entering *via* the airway (81). Nevertheless, pulmonary dendritic cells have been suggested to be the primary antigen presenting cells helping to activate an immune response (82).

There are several types of primary lung cancers which are characterized by distinct immune tumor microenvironments (83). Some lung cancers such as non-small lung cancer are also known to harbor specific driver mutations including EGFR and KRAS mutations (84). An immunohistochemistry-based study of NSCLC also found that most patients are clustered into a subtype classified as ‘immune activated’, characterized by high CD4+ T cells, CD8+ T cells and CD20+ B cells, which subsequently associated with longer disease-free survival in treatment naïve early stage resected NSCLC patients. However, they also observed 2 other clustered subsets of NSCLC patients that had increased expression of CD133 and FoxP3, and these were associated with shorter disease-free survival (85).

Lung metastases have been described as being more immunogenic, with an increased immune cell infiltration, An overview of the tumor immune microenvironment of lung metastases from reported studies has been depicted in **Figure 1**.

Our previous research has shown that melanoma lung metastases had a significantly higher CD3+ T cell density compared to other sites of metastases, including the liver and brain (73). Moreover, lung metastases had a higher proportion of PD-1+ T cells and of Tim-3+ T cells compared to other sites including the liver and brain, respectively (73). In this study, lung metastases also had a higher density of CD68+ macrophages as well as the highest PD-L1 expression on both tumor cells and macrophages compared to other sites of metastases. In the study by Ballas et al. assessing NK cells in the lung and liver melanoma metastases, whilst liver metastases had mainly CD27+ CD11b- NK cells, which were suggested to be protective in the development of metastases, lung metastases had a higher concentration of mature CD27- CD11b+ NK cells, which may not have as efficient protective mechanisms (75). Yu and Green et al. also showed that, in contrast to liver metastases, the presence of lung metastases did not alter the levels of T cells in other sites (59).

An immunophenoscore (IPS) has been developed to provide a scoring method across cancers using gene expression data which reflects the immunogenic state of a sample utilizing the expression and prevalence of checkpoint markers, effector and suppressor cells and antigen presenting cells (86). Clinical and gene expression data from distant metastases in patients across various cancer types including breast, colorectal, RCC, NSCLC, prostate, and melanoma showed that lung metastases have a higher immunophenoscore, and are more immunogenic through increased density of B cells, T cells, endothelial cells, myeloid dendritic cells and fibroblasts compared to other sites of metastasis (87). Patterns of immune cells present were also shown to remain constant in lung metastases irrespective of primary tumor type. A separate immunohistochemistry study supported that the number of T cells were similar in lung metastases regardless of if the patients had renal cell carcinoma or colorectal cancer. However, this study found that other immune cells including NK cells and dendritic cells were discordant in lung metastases depending on the primary cancer types (88). Specifically, this study found that lung metastases from patients with colorectal carcinoma had increased DC-LAMP+ dendritic cells but reduced NKp46+ NK cells compared to lung metastases from patients with RCC. Furthermore, this study showed that the same immune cell type might have a distinct role in different cancer types, as increased densities of DC-LAMP+ dendritic cells was associated with shorter OS in RCC patients but longer OS in colorectal carcinoma patients (88).

### 3.3 Brain metastases

#### 3.3.1 Clinical outcomes in patients with brain metastases treated with immunotherapy

In advanced cancer patients, the presence of brain metastases has been associated with reduced OS and thus associated with a bad prognosis. Patients with brain metastases have often been excluded from many clinical trials due to their poorer prognosis (89, 90). Thus, the availability of clinical response data is much more limited compared to other sites of metastasis. A summary of clinical studies assessing the local and systemic response of brain metastases are summarized in **Table 1**.

In melanoma, brain metastases have been associated with reduced PFS and OS (but not ORR) in immunotherapy treated patients (78, 91). A multicenter randomized phase 2 trial, the ABC trial, compared the efficacy of anti-PD-1 monotherapy and combination IPI+anti-PD-1 in melanoma patients with asymptomatic brain metastases and showed that patients treated with the combination achieved higher rates of response both intracranially (ORR = 46%, and ORR = 56% for drug treatment naïve) and extracranially (ORR = 57%, and ORR = 63% for drug treatment naive) compared to anti-PD-1 alone (ORR = 20% intracranially, and ORR = 21% for drug treatment naïve) and extracranially (ORR = 29%, and ORR = 29% for drug treatment naïve)) (92). The checkmate 204 trial further supports these data, showing that approximately half of melanoma patients with asymptomatic brain metastases responds to the combination IPI+anti-PD-1 (93).

A phase 2 trial specifically assessing the impact of anti-PD-1 monotherapy in NSCLC patients with brain metastases showed an ORR of 29.7% in the subset of patients whose tumor expressed PD-L1, suggesting some activity in NSCLC brain metastases (94). In a retrospective study of NSCLC patients with brain metastases, the addition of anti-PD-1 to whole brain radiotherapy (WBRT) showed better survival compared to WBRT alone (median OS, 27 versus 20 months) in patients with brain metastases (95). The extent to which brain metastases responds compared to other sites in NSCLC patients however is still to be explored.

Furthermore, in a small retrospective study, brain metastases were associated with the worst local response rates compared to other sites including lung, liver, adrenal gland, pancreas and lymph node in advanced RCC patients treated with single agent anti-PD-1 (96).

#### 3.3.2 Brain-specific tumor microenvironment

The brain is typically considered an immune privileged site; the unique blood brain barrier protects the brain from inflammation/immune cell infiltration. Therefore, the brain endothelial cells are uniquely packed tightly together, thus inhibiting the movement of cells from the blood circulation to the brain tissue (97, 98). The integrity of the blood brain barrier can be impaired through the course of some diseases (e.g. brain metastases), but there are brain resident cells, including astrocytes, that play an active role in maintaining permeability of the blood brain barrier in physiologic conditions (99). The brain also has tissue resident immune cells such as specialized macrophages of the brain, known as microglia, which play a major role in the innate immune response, including phagocytosis of cellular debris and antigen presentation (100).

Primary brain tumors have been shown to be comprised of a wide range of cellular components, a large portion of which are macrophages, both tissue resident and peripherally derived, including tumor associated macrophages, which are typically classified as pro-tumorigenic (101, 102). Neutrophils have also been shown to be prognostic for primary brain tumors in that increased intratumoral neutrophils are associated with high-grade tumors (102).

Brain metastases have been characterized as being less immunogenic with unique immune profiles compared to other sites of metastases, however some disparity in the findings between studies have been observed. An overview of the tumor immune microenvironment of brain metastases from reported studies has been depicted in **Figure 1**.

Davies et al. showed that melanoma brain metastases had increased activation of the PI3K/AKT pathway, particularly compared to unmatched liver and lung metastases. A follow-up study by Chen and Davies et al. that assessed oncogenic driver mutations, gene expression profiles and protein expression between matched brain and extracranial metastases showed that whilst oncogenic mutations and gene expression patterns were mostly conserved between matched samples, there was a specific increased activation of the PI3K/AKT pathway observed in brain metastases compared to extracranial metastases (103, 104). Our group have shown that melanoma brain metastases, similarly to liver metastases, also have a reduced density of intratumoral CD3+ T cells compared to other sites, like subcutaneous, lymph node and lung metastases (73). This is in line with previous published data by Kluger et al, which showed that brain metastases have reduced CD3+ T cell infiltration compared to extracranial metastases (105). Unlike liver metastases, however, brain metastases had an increased proportion of CD3+ T cells expressing PD-1 (73).

In a small cohort of metastatic NSCLC patients, Zhou et al. observed fewer CD8+ T cells in brain metastases compared to the primary lung lesions. Furthermore, they observed higher PD-L1 expression on tumor cells but not immune cells in brain metastases compared to primary lung lesions (106). A larger NSCLC study compared matched primary lung lesions and metastatic brain metastases, and whilst it supports a reduction in T cell infiltration in brain metastases compared to primary lung lesions, it also observed a reduction of PD-L1 expression at the metastatic site, including brain metastases, compared to the primary site (107). Another retrospective study comparing primary NSCLC tumors and matched brain metastases found that most immune cells were reduced in the brain. M2 macrophages and NK cells, however, were in higher densities in brain metastases compared to matched primary lung tumors (108). Kudo et al. also assessed the differences in the tumor immune microenvironment of the primary site compared to brain metastases in NSCLC. At the genomic level they found high concordance in the mutations between both sites, however differences were observed at both the transcriptomic and protein expression levels. Specifically, reduced T cells and dendritic cells (DCs) were observed in brain metastases compared to primary lung cancer. Furthermore, brain metastases had reduced extravasation signaling, DC maturation and expression of vascular cell adhesion molecule 1 (VCAM1) compared to the primary site, while there was an increase in arginase 1 (ARG1) suggesting an increase in M2-like macrophages in brain metastases compared to the primary lung tumor (109).

### 3.4 Bone

#### 3.4.1 Clinical outcomes in patients with bone metastases treated with immunotherapy

Bone metastases have been typically associated with a poor prognosis across many cancer types including prostate, breast, melanoma, NSCLC, colon, stomach, bladder, and thyroid cancer (110–113). A summary of clinical studies assessing the local and systemic response of bone metastases are summarized in **Table 1**.

In patients with metastatic melanoma treated with combination IPI+anti-PD-1, the presence of bone metastases has been associated with reduced ORR and shorter PFS compared to the presence of lung metastases (58). In a secondary analysis of patients treated with single agent anti-PD-1 on the Checkmate-003 trial, which included patients with NSCLC, melanoma and renal cell carcinoma, the presence of bone metastasis was associated with reduced OS (114). In contrast, a subgroup analysis of the Checkmate-025 trial of RCC patients randomized to either anti-PD-1 monotherapy or everolimus showed that patients with bone metastases had an OS benefit with immune checkpoint inhibitors compared to everolimus (median OS 18.5 months *vs.* 13.8 months) supporting the use of immune checkpoint inhibitors for bone metastases (115). Whilst patients with bone metastases have been included on many studies, there is limited analysis performed comparing patients with and without bone metastases to clearly understand the response of bone metastases compared to other sites when treated with immune checkpoint inhibitors, across different cancer types.

Landi et al. studied the impact of anti-PD-1 monotherapy on bone metastases in NSCLC patients (squamous and non-squamous). They observed that patients with metastatic squamous cell carcinoma of the lung with bone metastases had lower ORR (13% versus 22%) and shorter PFS (2.7 months versus 5.2 months) and OS (5.0 months versus 10.9 months), compared to patients without bone metastases (116). Similar observations were seen in the non-squamous NSCLC patients, also with poorer outcomes in patients with versus without bone metastasis (ORR, 12% versus 23%; PFS, 3.0 months versus 4.0 months; and OS, 7.4 versus 15.3 months) (116). Another study by Li et al. of NSCLC patients showed that when treated with immune checkpoints inhibitors in monotherapy, patients with bone metastases had shorter OS (median OS, 12.5 and 23.9 months) and shorter PFS (median PFS, 4.2 and 6.7 months) versus patients without bone metastases. Such a difference was not observed in patients treated with immune checkpoint inhibitors in combination with antiangiogenics or chemotherapy (117). Whilst bone metastases, like liver metastases, have been associated with a poor prognosis, the large retrospective study by Ren et al. found that patients with bone metastases as the only site of disease had a greater median OS compared to those with solitary liver metastases in both adenocarcinoma (5 months versus 3 months) and in small cell lung cancer patients (7 months versus 3 months). Overall, this study suggests that bone metastases was still associated with a better prognosis compared to liver metastases (57, 113).

#### 3.4.2 Bone-specific tumor microenvironment

Two of the most notable bone specific cells are osteoclast and osteoblasts. Osteoclasts play a major role in bone resorption whilst osteoblasts aid in bone formation primarily during remodeling and bone development (118). Osteoblasts under normal conditions are known to produce a large number of growth factors and enzymes including type 1 collagen and TGFβ to aid in bone formation. Osteoblasts also further differentiate into osteocytes in fully formed bone (119).

Osteosarcoma is one of the most common primary cancers of the bone. Studies delving into the tumor microenvironment of primary bone cancer however faces its challenges. Osteosarcoma has a complex and heterogenous microenvironment including the presence of fibroblasts and other stromal cells, bone cells, pericytes and vascular cells and other infiltrating immune cells including T cells, B cells, NK cells and dendritic cells (120). The heterogeneity of osteosarcoma has also been attributed to genomic alterations including chromosomal aneuploidy, copy number variations and genomic instability as a result of chromotripsis (121).

Bone metastases have been characterized as harboring a unique tumor immune microenvironment, particularly in the interaction between bone specific immune cells and the tumor. Specifically, an increase in osteoclast activity and the RANKL pathway have been shown to increase tumor cell invasion and growth. An overview of the tumor immune microenvironment of bone metastases from reported studies has been demonstrated in **Figure 1**.

The earlier highlighted study by García-Mulero et al. showed that, like lung metastasis, bone metastases showed an increase in stromal cells and fibroblasts compared to other sites including the liver and brain. While lung metastases appear concordant in immune cell populations regardless of primary lesion type (as described above), bone metastases differed depending on the primary tumor location; there was an increase in antigen presentation and effector cells in renal cell carcinoma and colorectal cancer compared to melanoma and prostate cancer bone metastases (87). Tumors in the bone have been shown to shift the bone microenvironment towards being more osteoclast dominated leading to increased bone resorption (122, 123). Specifically, the pre-clinical murine study by Jiao et al. of castrate resistant prostate cancer bone metastases showed that there was increased osteoclast activation in tumor bearing bones, with subsequent increase of TGF-β1 expression (124). Increased excretion of mediators such as TNF-a, prostaglandin, IL-1 and IL-6 by bone metastases have also been shown to increase osteoclast activation *via* the receptor-activator of nuclear kappaB ligand (RANKL) pathway, leading to further bone resorption (110, 123). Increased bone resorption has also been shown to play a role in the vicious cycle where aside from increasing activation of the RANKL pathway, TGFβ and IGF released from increased bone resorption instead of being used by osteoblasts in bone formation is able to increase tumor cell growth and proliferation (119). A murine and cell culture study by Lu et al. whereby bone metastases were established in mice by intracardiac injections showed an increased VCAM-1 expression in tumor cells was associated with recruitment and activation of osteoclasts promoting osteoclastogenesis and allowing for increased bone metastatic growth (125).

Targeted immunohistochemistry assessment by Akfirat et al. of prostate cancer metastases showed that prostate cancer bone metastases had increased expression of MCL-1 (myeloid leukemia 1) and BCL-2 (B-cell lymphoma 2), key contributors in cell survival, compared to other sites of metastases (126–128). In a murine model of lung cancer with bone metastases, Wang et al. showed that bone metastases had an upregulation of PD-L1 and CCL2 (chemokine ligand 2), and that this increased expression induced the formation of osteoclasts *via* the RANKL pathway (129). The study of the immune microenvironment of bone metastases is challenging due to low sample availability, bone destruction and difficulty in preparing samples for experiments and analysis due to the bone structure.

### 3.5 Other sites of metastases

#### 3.5.1 Clinical outcomes in patients with other sites of metastases treated with immunotherapy

Whilst metastatic sites such as liver, lung, brain, and bone are some of the most common sites of metastasis for many cancers, other metastatic sites may include lymph nodes, skin/soft-tissue, muscle, adrenal gland, gastrointestinal tract and peritoneum (130–133). However, there is limited data available assessing the response of these other sites of metastasis, and the data is generally restricted to case reports and case series. A summary of clinical studies assessing the local and systemic response of other sites of metastases are summarized in **Table 1**.

Subgroup analysis of previously treated metastatic TNBC patients in the keynote-086 trial showed that there was an increased ORR in patients who had lymph node only metastases (ORR, 27.8%) versus metastases to other sites (ORR, 2.6%) when treated with single agent anti-PD-1 (65). In keeping with these findings, the retrospective study by Schmid et al. of metastatic NSCLC patients treated with anti-PD-1 monotherapy also showed that the OSRR was greater in lymph node metastases (OSRR = 28%) compared to other sites, including liver and lung metastases (134). This study also assessed adrenal metastases (OSRR = 33%) and soft-tissue metastases (OSRR = 0%), however the numbers of metastases at these sites were too small to accurately interpret the findings. Further, another retrospective study assessed adrenal gland metastases in melanoma patients treated with immune checkpoint inhibitors. This study found that adrenal gland metastases had reduced disease control rate (complete response [CR] + partial response [PR] + stable disease [SD]; DCR = 29%) compared to other sites including liver, lung and brain (DCR = 46%). It was shown that, in melanoma patients treated with anti-PD-1, those with adrenal gland metastases had reduced overall survival (median OS, 2.7 years) versus those without adrenal gland metastases (median OS, not reached; HR 3.12) (135). In contrast, a separate retrospective study of previously treated NSCLC patients enrolled onto the phase III OAK trial aimed at comparing anti-PD-L1 therapy versus chemotherapy, showed that in site-specific pair wise comparisons, adrenal gland metastases were associated with greater overall survival in immunotherapy treated patients compared to those with other sites of metastasis including liver and bone (136).

#### 3.5.2 Other site-specific tumor microenvironments

Whilst non-metastatic lymph nodes are areas of high immune cell infiltration, understanding the immune microenvironment once metastases are established may play a key role in helping dissect their response to immune checkpoint inhibitors. A study by Popeda and colleagues comparing transcriptional changes between breast cancer primary tumors and lymph node metastases found that the metastases had an upregulation of ATG10 (autophagy related 10), S100B (S100 calcium-binding protein B), and GATA3 (GATA binding protein 3), whilst a decrease in the expression of 33 genes was found, most of which were associated with innate immunity and the complement cascade (137). An immunohistochemistry study by Kim et al. comparing primary squamous cell carcinoma of the lung and respective lymph node metastases found that the expression of PD-L1 and PD-L2 on tumor cells were, in most cases, conserved between primary tumors and lymph node metastases. PD-L1 and PD-L2 expression also positively correlated with CD8+ T cells and PD-1+ T cell density in this cohort suggesting the number of CD8+ T cells and PD-1+ T cells were also conserved between primary tumors and lymph node metastases (138). Studies undertaken by our own group also observed that in treatment naïve advanced melanoma patients, lymph node metastases had higher CD3+ T cell density compared to other sites, particularly liver and brain metastases. Further, whilst making up a small proportion, lymph node metastases, along with subcutaneous metastases, had increased FoxP3+ T cells compared to other sites (73). In this same study, subcutaneous metastases, however, were observed to have the highest proportion of CD103+ T cells.

Wang et al. performed genomic sequencing of matched gastric cancer primary tumors and peritoneal metastases, and observed that inactivating TAF1 and CDH1 mutations were more prevalent in the peritoneal metastases compared to the primary lesions. Further transcriptomic data also showed a subset of peritoneal metastases termed as “T-Cell exhausted” as being characterized by increased expression of Tim3, Galectin-9 and VISTA (139).

Whilst studies into the tumor immune microenvironment of adrenal metastases is limited, a small study of matched primary lung cancer lesions and adrenal metastases observed an increased expression of CCR6, chemokine responsible for the recruitment of immune cells (e.g. DCs, effector T cells and B cells) in the metastatic lesions compared to the primary lesion (140).

## 4 Concluding remarks

Traditionally, treatment regimens have been selected based on tumor type, staging and other prognostic factors. The pattern of metastases has not been a major factor in therapy choice for most cancers and largely overlooked. However, as highlighted in this review, the presence or absence of specific sites of metastasis is not only prognostic but also impact response in patients treated with immune checkpoint inhibitors. Whilst recent studies have highlighted the influence of specific sites of metastasis on the response to immune checkpoint inhibitors, the biological mechanisms underlying these clinical observations is still unknown. In this review, we have highlighted organ-specific responses to immune checkpoint inhibitors, particularly focusing on the lung, liver, bone and brain, with the latter three sites associated with a poor response across multiple primary cancer types. This further highlights the importance of considering the site of metastasis when choosing treatment options and not just the cancer type.

Metastases at various sites present distinct tumor immune microenvironment, not only between different organ sites, but also between the primary tumor and the metastatic sites. Whilst data is still somewhat limited, further studies into the tumor immune microenvironment may provide insights into novel organ specific mechanisms of resistance that can be targeted with new therapeutic options. Nevertheless, assessing and comparing specific tumor immune microenvironments in humans faces challenges. Obtaining pre-treatment tissue samples from multiple sites from the same patients is a notable limitation. Matched patient samples are often obtained from autopsy programs when patients have already been heavily treated.

In summary, whilst the differences in organ-specific response to immune checkpoint inhibitors and the tumor immune microenvironment is emerging as an important factor in therapy efficacy, there is still limited data and a lack of understanding of the biological mechanisms. Further research into this emerging important area is urgently needed. Larger focus on assessment of these differences may help elucidate organ-specific resistance mechanisms and is crucial in providing more personalized treatment approaches.

## Author contributions

Manuscript design and conception: JC and IP. Manuscript draft: JC and IP. Review and revision of manuscript: JB, JW, RS, and GL. Supervision: RS, GL, and IP. All authors contributed to the article and approved the submitted version.

## Funding

This work was supported by a National Health and Medical Research Council of Australia (NHMRC) Program Grant (APP1093017) (to RS and GL). JC is supported by the Emma Betts MIA PhD Scholarship and an Australian Melanoma Research Foundation (AMRF) Post-Graduate research grant. JW and GL are supported by NHMRC Investigator Grants. GL is also supported by the University of Sydney Medical Foundation. RS is supported by an NHMRC Practitioner Fellowship (APP1141295). IP is supported by a Cancer Institute NSW Early Career Fellowship. Support from the CLEARbridge Foundation, and the Cameron Family is also greatly acknowledged.

## Acknowledgments

**Figure 1** was created with BioRender.com. Ongoing support from colleagues at the melanoma Institute Australia and the Charles Perkins Centre, University of Sydney is also greatly acknowledged.

## Conflict of interest

GL is consultant advisor for Agenus, Amgen, Array Biopharma, Boehringer Ingelheim, Bristol Myers Squibb, Evaxion, Hexal AG Sandoz Company, Highlight Therapeutics S.L., Innovent Biologics USA, Merck Sharpe & Dohme, Novartis, OncoSec, PHMR Ltd, Pierre Fabre, Provectus, Qbiotics, Regeneron. RS has received fees for professional services from F. Hoffmann-La Roche Ltd, Evaxion, Provectus Biopharmaceuticals Australia, Qbiotics, Novartis, Merck Sharp & Dohme, NeraCare, AMGEN Inc., Bristol-Myers Squibb, Myriad Genetics and GlaxoSmithKline. IP had travel support by BMS and MSD, and speaker fee by Roche, Bristol-Myers Squibb and Merck Sharpe & Dohme.

The remaining authors declare that the research was conducted in the absence of any commercial or financial relationships that could be construed as a potential Conflict of interest. The handling editor MA declared a past co-authorship with the author GL.

## Publisher’s note

All claims expressed in this article are solely those of the authors and do not necessarily represent those of their affiliated organizations, or those of the publisher, the editors and the reviewers. Any product that may be evaluated in this article, or claim that may be made by its manufacturer, is not guaranteed or endorsed by the publisher.
